# Setup of a Simple and Cost-Effective pH-Sensitive Assay to Evaluate Phagocytosis in Rainbow Trout (*Oncorhynchus mykiss*) Peripheral Blood Leukocytes

**DOI:** 10.3390/ani16121760

**Published:** 2026-06-06

**Authors:** Teresina De Iorio, Maria Carmela Scatà, Arianna Martini, Marco Martinoli, Riccardo Napolitano, Nicolò Tonachella, Domitilla Pulcini, Fabrizio Capoccioni

**Affiliations:** 1 Consiglio per la Ricerca in Agricoltura e l’Analisi dell’Economia Agraria (CREA), Centro di Ricerca Zootecnia e Acquacoltura, Via Salaria, 31, 00015 Monterotondo, RM, Italy; arianna.martini@crea.gov.it (A.M.); marco.martinoli@crea.gov.it (M.M.); riccardo.napolitano@stir.ac.uk (R.N.); n.tonachella@gmail.com (N.T.); domitilla.pulcini@crea.gov.it (D.P.); fabrizio.capoccioni@crea.gov.it (F.C.); 2 Institute of Aquaculture, University of Stirling, Stirling FK9 4LA, UK

**Keywords:** rainbow trout, phagocytosis, opsonization, innate immunity, fish health

## Abstract

Phagocytosis *in vitro* assays are widely used to assess the health status of fish, since it may be affected by diseases and environmental factors. To date, studies have evaluated phagocytosis in fish by observing fluorescent beads or bacteria engulfed by phagocytes. However, particles adhering to the external surface of the phagocyte membrane cannot be easily distinguished from those that are truly internalized. This work aimed to: (1) test whether a pHrodo™ (pHR)-based method improves the accuracy of phagocytosis detection compared to traditional assays; (2) evaluate the influence of opsonization, incubation time, and temperature on the phagocytosis ability of rainbow trout (*Oncorhynchus mykiss*) peripheral blood leukocytes (PBLs). Opsonization increased the PBLs’ phagocytic activity. To evaluate the effects of incubation time and temperature on PBL phagocytosis ability, 30% autologous plasma was used. Leukocytes incubated at 16 °C for 2 h showed a significantly higher (*p* < 0.05; H = 45.43) percentage of phagocytic cells. This incubation setting also resulted in increased pHR-related fluorescence intensity (*p* < 0.05; F = 58.39), directly proportional to the number of phagocytosed particles. These findings indicate that opsonization enhances phagocytic ability and capacity, and that a 2 h incubation at 16 °C may be recommended for future large-scale studies aimed at evaluating innate immune function in rainbow trout.

## Simple Summary

Phagocytosis is a key defense mechanism in which immune cells engulf and destroy bacteria and other harmful particles. Traditionally, researchers have assessed phagocytosis by observing fluorescent beads or bacteria that phagocytes internalize to estimate fish health and welfare. However, commonly used fluorescent particles remain visible even when attached only to the cell surface, making it difficult to accurately measure phagocytosis *in vitro*. This study evaluated a method based on pHrodo™-conjugated *E. coli*, a fluorescent bacteria-conjugated probe that produces a signal only when bacteria are taken up and broken down inside immune cells. The aims were: (1) to test whether this method could improve the accuracy of phagocytosis detection compared with traditional fluorescent particles; (2) to assess optimal in vitro conditions for rainbow trout (*Oncorhynchus mykiss*) leukocytes. The rainbow trout was used as an experimental model to examine how factors such as coating bacteria with blood proteins (opsonization), incubation time, and temperature affect the assay. The results identify optimal conditions for this assay and provide a useful tool for future studies on rainbow trout immune system, which may support research aimed at improving animal health in aquaculture.

## 1. Introduction

Over the last decade, fish immunology has gained increasing attention, as the expanding aquaculture industry requires reliable methods for assessing disease risk and associated immune responses [1,2,3,4,5]. In this context, advancing knowledge of immune mechanisms in teleost fish is not only economically relevant but also essential for promoting environmentally responsible and ethically sound farming practices [5].

Despite significant progress, important gaps remain in the standardization and reproducibility of immunological assays in fish. Functional immunological tests are essential to evaluate the immune system’s response to various stimuli [6]. However, such research requires reliable *in vitro* assays and methods to assess both leukocytes’ innate and adaptive immune functions [7]. The lack of harmonized protocols still represents a major limitation, hindering meaningful comparisons across studies, laboratories, and species [8].

Phagocytosis is considered a crucial defense mechanism, since it contributes to pathogen clearance and to maintain overall tissue homeostasis [9,10]. This process involves myeloid leukocyte populations, and may be affected by diseases and environmental factors [11]. In aquaculture settings, exogenous stressors, such as temperature fluctuations, handling procedures, and variations in water quality, are known to modulate immune efficiency, including phagocytic activity [12]. Therefore, phagocytosis assays are widely used to assess fish health and welfare [13].

To date, phagocytosis in fish has primarily been evaluated by observing fluorescent particles being engulfed by phagocytes to assess their activity *in vitro*. The most commonly used methods employ zymosan (isolated from yeast cell walls), bacteria, and/or polystyrene/latex beads [10,13,14,15,16]. However, particles adhering to the external surface of the phagocyte membrane cannot be easily distinguished from those that have been internalized, leading to an inaccurate estimation of innate immune function [17]. To overcome this limitation, in recent years, pH-sensitive dyes (CypHer5E, TPE-Cy, and pHrodo™) have been developed [13]. The fluorescence of these dyes is absent at neutral pH and increases sharply as the pH decreases, allowing the analysis of the phagolysosome-internalized particles [18,19]. This property significantly reduces false-positive signals and improves the accuracy of phagocytosis measurements [20]. While these technologies have been extensively validated in mammalian cells in vitro, their application in fish immunology remains limited and requires further optimization.

Several methods have been developed to measure phagocytosis, including microscopy and flow cytometry approaches. Microscopy-based methodologies are particularly time-consuming. However, they enable direct visualization of cell-to-particle interactions, providing morphological information and qualitative insight into particle localization. In contrast, flow cytometry-based techniques enable rapid and reproducible detection of single-cell behavior. However, distinguishing membrane-bound from ingested targets using flow cytometry and conventional fluorescence microscopy remains challenging [17,18].

Additionally, experimental conditions such as incubation time, temperature, and opsonization protocols vary widely across studies. They can profoundly influence assay outcomes, leading to inconsistent results, complicating data interpretation [21,22,23] and highlighting the need for optimization [10]. Therefore, the present study aims to contribute to the standardization and methodological refinement of phagocytosis assays in fish. Specifically, we evaluate a pHrodo™-based approach to investigate the effects of opsonization, incubation time, and temperature on the phagocytic activity of rainbow trout (*Oncorhynchus mykiss*) peripheral blood leukocytes using conventional fluorescence microscopy and flow cytometry. Rainbow trout was selected as a model species since it is the most widely farmed freshwater fish in Italy and Europe, both in terms of production volume and economic value, representing a key species for the aquaculture sector. However, the proposed approach may be applied to a broader range of farmed species, including both fishes [17] and bivalve mollusks [17,24].

By systematically addressing key experimental variables, this work seeks to improve the reliability, sensitivity, and comparability of phagocytosis measurements, thereby providing a valuable tool for both basic research and applied studies in fish immunology and aquaculture.

## 2. Materials and Methods

### 2.1. Ethical Statement

This study complies with the European Union (EU) Directive 2010/63/EU on the protection of animals used for scientific purposes. All procedures included in this study were performed in accordance with national laws and institutional guidelines and were approved by the committee responsible for animal welfare at the CREA Animal Production and Aquaculture Research Centre (Authorization number: 00017431 of 29 February 2024).

### 2.2. Rainbow Trout Farming Conditions and Peripheral Blood Sampling

For this study, nine rainbow trout (*Oncorhynchus mykiss*) weighing 405 ± 66 g were reared in a rectangular earth pond in a commercial farm (Rieti, Central Italy), with an average water temperature of 16 ± 0.2 °C, pH of 8 ± 0.2, and 9.4 ± 0.1 mg/L of dissolved oxygen. Trout were fed twice a day with a commercial pellet diet (Optiline 3P, Skretting, Stavanger, Norway). Selected rainbow trout were randomly caught using a fishing net and euthanized by the farmer, according to the national law. Subsequently, 4 mL of peripheral blood was collected from the caudal vein through a luer-lock adapter 21 G butterfly needle (Beckton Dickinson, Franklin Lakes, NJ, USA) connected to vacutainer tubes containing Li-Heparin (Beckton Dickinson, Franklin Lakes, NJ, USA).

### 2.3. Isolation of Leukocytes from Rainbow Trout Peripheral Blood

Peripheral blood leukocytes (PBLs) were isolated as previously described [8]. Briefly, 4 mL of blood was transferred to a 50 mL tube, containing 36 mL of cell culture-grade water, and mixed by inversion for 20 s to lyse erythrocytes. Four milliliters of 10× Dulbecco’s Phosphate-Buffered Saline (DPBS, BioConcept, Allschwil, Switzerland) was then added to restore isotonicity. The resultant suspension was put on ice for 10 min to allow the sedimentation of cell debris and nuclear material. PBLs were purified by passing the suspension through a 100 µm cell strainer (Sarstedt, Nümbrecht, Germany) and pelleted by centrifugation at 300× *g* for 5 min. Cells were washed once with 40 mL Leibovitz medium (L-15; Gibco, Grand Island, NY, USA) supplemented with 1% fetal bovine serum (FBS; Gibco, Grand Island, NY, USA). Finally, PBLs were counted with LUNA^TM^ automated cell counter, and resuspended at a final concentration of 1 × 10^6^ cells/mL in a complete medium, composed of Leibovitz’s 15 medium (L15, Gibco) supplemented with 10% FBS, 1× antibiotic/antimycotic solution (Gibco), and 2 mM L-Glutamine (Sigma-Aldrich, St. Louis, MO, USA).

### 2.4. Evaluation of Phagocytosis in Rainbow Trout Peripheral Blood Leukocytes

#### 2.4.1. FluoSpheres™ Polystyrene Microspheres, 1.0 μm, Yellow-Green Fluorescent

A single polystyrene bead suspension (Thermo Fisher, Waltham, MA, USA) was prepared by sonicating the solution with a water-chilled ultrasonic sonicator for 30 s ×3 (300 ultrasonik, Ney, Brussels, Belgium). Then, a 20:1 particle-to-cell ratio was added to 1 × 10^6^ PBL/mL in complete medium (see paragraph above), and the mixture was co-incubated for 2 h at 16 °C. Cells were washed in 1X DPBS supplemented with 1% Bovine Serum Albumin (BSA; Sigma-Aldrich, St. Louis, MO, USA), centrifuged at 300× *g* for 5 min, and resuspended in complete medium. At the end of the incubation, the PBLs were rinsed in DPBS and either analyzed directly (Control, Ctr), stratified over a density gradient (Cushion), or subjected to Trypan blue quenching. Briefly, to perform a density gradient separation, cell suspension was stratified over a 3% BSA, 4.5% D-Glucose cushion and centrifuged at 100× *g* for 10 min, without brake, to remove non-internalized particles, as described by [14,25]. To quench the fluorescence emission of non-internalized beads, trypan blue solution was added, as described by [15,26]. Thereafter, samples were washed once with DPBS supplemented with 1% BSA and pelleted by centrifugation (300× *g* for 5 min at 4 °C).

#### 2.4.2. *Escherichia coli* (K-12 Strain) BioParticles™, Fluorescein Conjugate

Fluorescein-conjugated *E. coli* (K-12 strain) BioParticles™ (Invitrogen, Carlsbad, CA, USA) solution was immersed in a water-chilled ultrasonic sonicator for 30 s × 3 (300 ultrasonik, Ney) to obtain a single-bacteria suspension. Then, the bacterial suspension was opsonized with 10%, 30%, or 60% autologous plasma from a common batch [8] in PBS for 60 min at room temperature. Opsonized *E. coli* was then centrifuged at 800× *g* for 10 min at 4 °C to remove plasma residuals and resuspended in DPBS. PBLs were co-incubated with a 20:1 particle/cell ratio of opsonized *E. coli* for 2 h at 16 °C in complete medium, washed once with 1% BSA in DPBS, and pelleted by centrifugation (300× *g* for 5 min at 4 °C).

#### 2.4.3. pHrodo™ Green *E. coli* BioParticles™ Conjugate for Phagocytosis

To obtain a single-bacteria suspension, the pHrodo™ Green Conjugated *E. coli* Bioparticles™ (Thermo Fisher Scientific, Waltham, MA, USA) were immersed in a water-chilled ultrasonic sonicator for 30 s × 3 (300 ultrasonik, Ney). The bacterial suspension was incubated with 10%, 30%, or 60% autologous plasma from a common batch [8] in DPBS for 60 min at room temperature, centrifuged at 800× *g* for 10 min at 4 °C. Opsonized pHrodo-*E. coli* were then resuspended in DPBS to evaluate the optimal plasma concentration for pHrodo™ Green *E. coli* BioParticles™ (pHR). Then, PBLs (1 × 10^6^/100 μL) were co-incubated with a 20:1 particle-to-cell ratio of either non-opsonized or opsonized pHR. Once the optimal plasma concentration was assessed, the influence of time and temperature on rainbow trout PBL phagocytosis was analysed by co-incubating PBL with opsonized pHrodo™ Green *E. coli* (20:1 particle/cell ratio) at 4 °C, 16 °C, or 26 °C, for one, two, or three hours.

For the microscopic analysis, cells were deposited onto a glass slide, fixed in 4% formaldehyde for 10 min, and mounted with ProLong™ Gold antifade mounting medium containing 4′,6-diamidino-2-phenylindole (DAPI; Thermo Fisher Scientific, Waltham, MA, USA). Samples were observed at 400× magnification under an Axioplan microscope (Zeiss, Oberkochen, Germany) equipped with a CMOS digital camera (Hayer, Tokyo, Japan). Images were taken with constant exposure parameters. A total of 15 randomly selected fields at 400× total magnification were analyzed for each specimen. Fluorescence intensity was evaluated by measuring particle intensity with ImageJ analysis software v 1.54 (https://imagej.net/ij/, last accessed on 1 January 2025) and expressed as Relative Fluorescence Units (RFU). PBL phagocytosis was estimated by counting cells and positive cells using the ImageJ Cell Counter plugin and expressed as a percentage. Lymphoid and myeloid cells were defined according to their nuclear morphology: small cells with rounded nuclei were considered lymphoid; large cells with either indented, half-moon, or multilobed nuclei were defined as myeloid.

For flow cytometry analysis, leukocytes were collected on a CytoFLEX flow cytometer (Beckman Coulter, Brea, CA, USA) using 488-nm excitation, and fluorescence emission was detected with a 525/40 BP filter. At least 100,000 leukocytes were measured after gating according to the FCS/SSC characteristics. Kaluza Analysis software v 2.1 (Beckman Coulter) was used to analyze the flow cytometric data. Dot plots of forward scatter (FSC) vs. side scatter (SSC) were set up to select lymphoid and myeloid cells by their size, as previously described [8].

### 2.5. Statistical Analysis

Fluorescence intensity and the percentage of positive cells, obtained by microscopy and flow cytometry, were presented as boxplots, with the box representing the interquartile range (IQR), bounded by the 25th and 75th percentiles; the horizontal line indicates the median, and the central point indicates the mean. Differences among experimental groups were assessed using the Kruskal–Wallis test, with Bonferroni correction for multiple comparisons. Differences of *p* ≤ 0.05 were considered statistically significant and are indicated by different superscripts. All the statistical tests were performed using the open-source software Past v. 4.14 (see https://palaeo-electronica.org/2001_1/past/pastprog/index.html, accessed on 1 May 2026) [27].

## 3. Results

The first set of experiments was designed to evaluate in vitro the influence of the assay used to estimate rainbow trout PBL phagocytic ability. Specifically, particular attention was given to identifying potential technical limitations associated with each methodological step to better interpret the reliability and accuracy of the obtained measurements.

**Figure 1:**
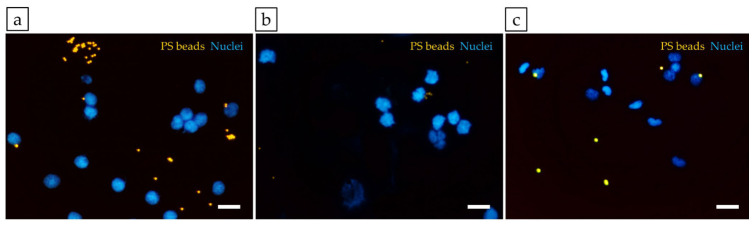
Representative fluorescence microscopic images showing Polystyrene (PS) bead-based assays used to study fish innate immune functions. Yellow PS beads incubated with rainbow trout PBL (Ctr; (**a**)), after trypan blue quenching (**b**), and with BSA cushion (**c**). Cell nuclei were stained with DAPI (blue). Scale bar: 10 µm.

Polystyrene bead assay showed highly fluorescent non-internalized and potentially internalized particles (Figure 1a). Residual fluorescence potentially leads to an overestimation of phagocytic activity. The trypan blue quenching step does not result in a complete signal quenching of non-internalized particles (Figure 1b). Moreover, the gradient cushion results in an incomplete removal of the unbounded beads (Figure 1c). The incomplete separation contributes to a background signal, highlighting the need for improved washing or separation procedures to enhance assay specificity.

**Figure 2:**
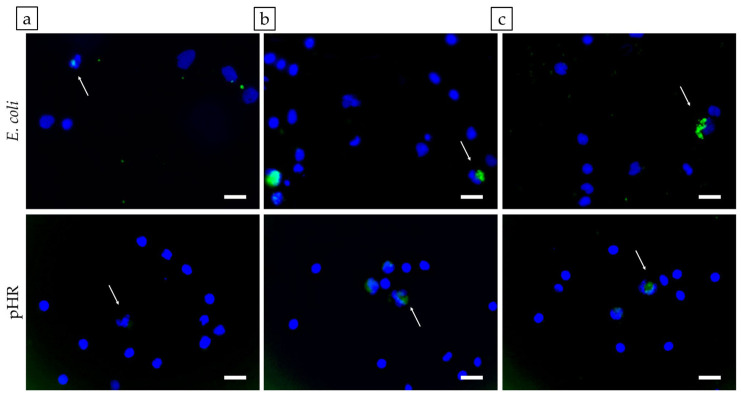
Representative fluorescence microscopic images showing bacteria-based phagocytosis assays used to study fish innate immune functions. Green fluorescent *E. coli* (top panel) and pHrodo-conjugated *E. coli* (bottom panel) phagocytosis assay, non-opsonized (**a**), opsonized with 30% (**b**) or 60% (**c**) of autologous plasma. Cell nuclei were stained with DAPI (blue), and phagocytic cells are highlighted with a white arrow. Scale bar: 10 µm.

In the bacterial-based assays, a pre-test was performed with different concentrations of autologous plasma to determine the optimal plasma concentration for the leukocyte phagocytosis assay. Bacteria opsonization significantly increased the amount of internalized *E. coli* (Figure 2a, top panel). However, membrane-bound particles cannot be easily distinguished from those internalized by conventional fluorescence microscopy, regardless of the opsonization concentration (Figure 2b,c, top panel). This limitation highlights a major drawback of traditional fluorescence-based approaches: they may overestimate phagocytic activity by including the surface-adhered bacteria in the analysis. In contrast, the pHRodo-conjugated *E. coli* (pHR) displayed higher specificity and sensitivity, allowing discrimination between internalized (fluorescent) and non-internalized bacteria (non-fluorescent) regardless of the opsonization (Figure 2a–c, bottom panel; Figure S1). Indeed, subtle green fluorescence observed in non-opsonized samples likely reflects non-opsonic phagocytosis. Although non-opsonized conditions may reveal basal phagocytic activity, they do not accurately reflect the in vivo situation, where circulating antibodies and complement factors continuously promote opsonization [28,29]. The specific property of the dye improved the assay robustness, which makes it fluorescent in the acidic environment of phagolysosomes, thereby providing a more accurate assessment of true phagocytic events. In addition, opsonization provides a more physiologically relevant and sensitive readout.

**Figure 3:**
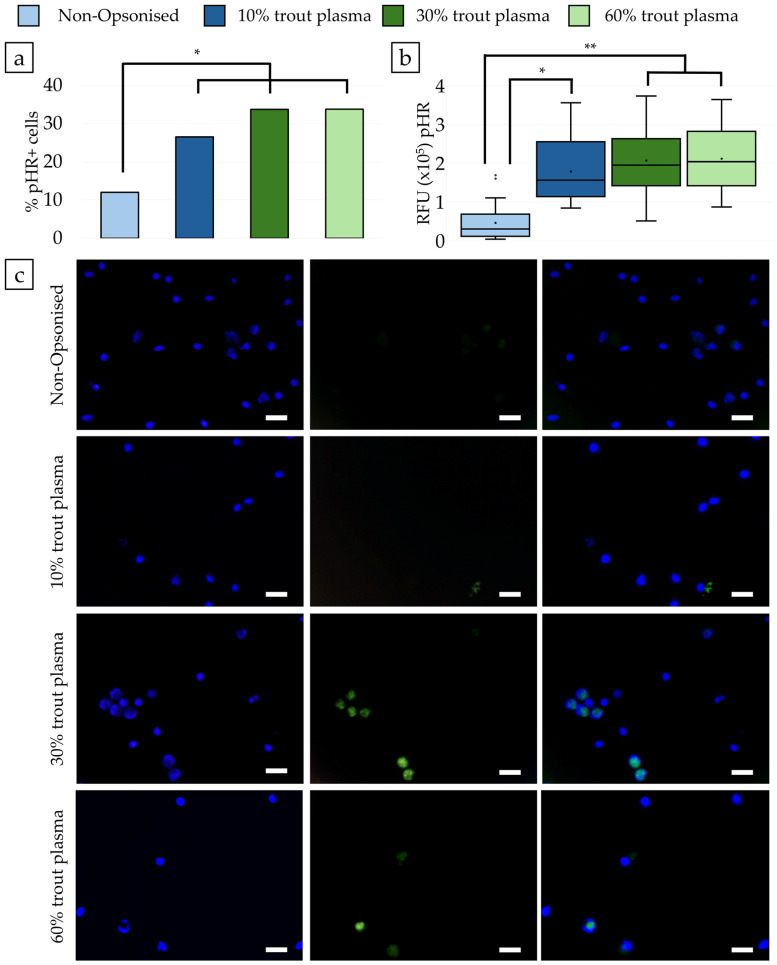
Effects of opsonization with 10%, 30%, and 60% autologous plasma on the phagocytosis ability of rainbow trout (*Oncorhynchus mykiss*) PBLs. Percentage of pHR+ cells (**a**), pHR-associated Relative Fluorescence Unit (RFU) (**b**), representative fluorescence microscopic images showing phagocytosis in non-opsonized and opsonized pHR-conjugated *E. coli* with 10%, 30%, and 60% of autologous plasma (**c**). Different asterisks denote statistically relevant differences (* *p* < 0.05, ** *p* < 0.01). Scale bar: 10 µm.

The image analysis revealed that opsonization sharply increased the percentage of phagocytic leukocytes, regardless of the plasma concentration used (Figure 3a,c). However, significantly higher fluorescence intensity was observed at the highest opsonin concentrations tested (30% and 60%, *p* < 0.01) compared with non-opsonized samples (Figure 3b). The increase in signal intensity reflects a greater number of bacteria internalized per cell, indicating that while the proportion of phagocytic cells remains stable, the efficiency of phagocytosis is enhanced at higher opsonin concentrations.

**Figure 4:**
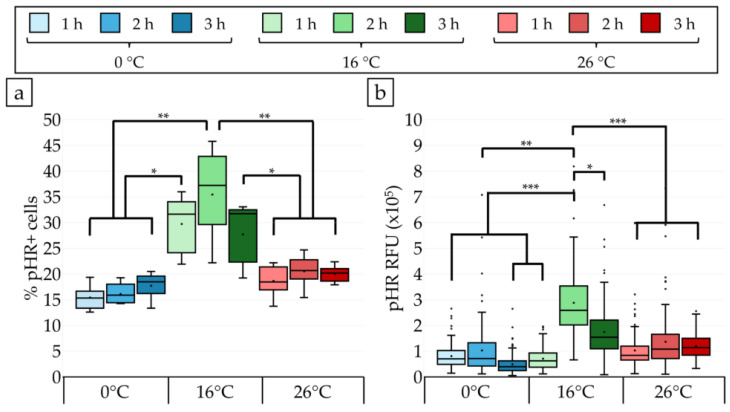
Microscopic evaluation of incubation temperature and time on the percentage (**a**) and Relative Fluorescence Unit (RFU) (**b**) of pHR+ myeloid subpopulation in rainbow trout (*Oncorhynchus mykiss*) PBLs. Different asterisks denote statistically relevant differences (* *p* < 0.05, ** *p* < 0.01, *** *p* < 0.001).

The second set of experiments aimed to evaluate the influence of time and temperature on the pHR-based phagocytosis assay in rainbow trout PBLs. To this end, nine different incubation conditions were systematically tested to identify the most suitable combination for subsequent experimental applications. The assessment of optimal time and temperature conditions showed a higher percentage of phagocytic PBLs at 16 °C, regardless of incubation time (Figure 4a). However, 2 h of incubation at 16 °C resulted in a significantly higher number of phagocytic cells (*p* < 0.01). Moreover, PBLs incubated at 16 °C for 2 h showed higher fluorescence from phagocytosed particles than the other conditions (Figure 4b).

**Figure 5:**
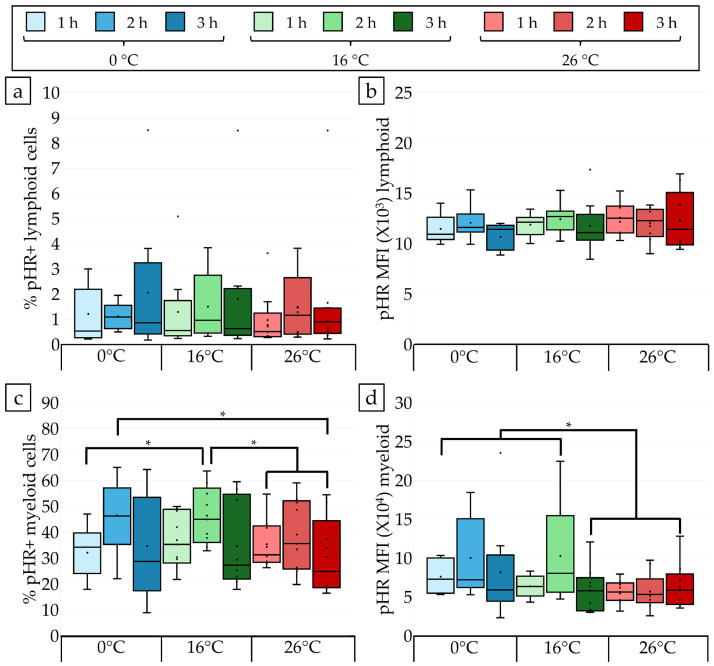
Results of flow cytometric analysis showing the effects of incubation temperature and time on the percentage of positive cells (**a**,**c**) and mean fluorescence intensity (MFI). MFI (**b**,**d**) of pHR+ lymphoid (**a**,**b**) and myeloid subpopulation (**c**,**d**) in rainbow trout (*Oncorhynchus mykiss*) PBLs. Different asterisks denote statistically relevant differences (* *p* < 0.05).

To confirm these results in a more efficient and higher-throughput method, flow cytometry analyses were also performed. Flow cytometry enhances the analytical power of trout leukocyte studies by enabling the discrimination, based on physical parameters, of phagocytic lymphocytes from phagocytic monocytes and neutrophils, thereby providing greater resolution and quantitative accuracy than microscopy; moreover, it allows the simultaneous evaluation of the phagocytic activity (represented by percentages of phagocytic cells) as well as their phagocytic capacity (represented by mean fluorescence intensity (MFI), which reflects the number of bacteria ingested). No significant differences were detected in the percentage or fluorescence intensity of phagocytic lymphoid cells across the tested conditions (Figure 5a,b). In contrast, the myeloid subpopulation incubated at 0 °C and 16 °C for 2 h showed a significantly higher percentage of pHR^+^ cells compared to cells incubated at 0 °C for 1 h or at 26 °C for 1 and 3 h (Figure 5c). These incubation settings also resulted in higher pHR-related MFI compared to 16 °C for 3 h and all conditions tested at 26 °C, regardless of incubation time (Figure 5d).

## 4. Discussion

This study assessed the limitations of the most commonly used phagocytosis assay for estimating innate immunity in aquatic animals using conventional fluorescence microscopy and optimized conditions for a pH-sensitive assay for measuring phagocytic activity in rainbow trout *in vitro*. We gave particular attention to methodological variables known to influence assay performance, including incubation time, temperature, and opsonization, all of which vary substantially across published studies and may compromise comparability and reproducibility [10,21,23]. Indeed, variations in temperature, incubation time, and opsonization protocols may yield inconsistent results, highlighting the need for methodological optimization. Therefore, our findings address an important need for standardized, biologically relevant protocols for teleost immunology.

To date, phagocytosis in fish has been mainly assessed using fluorescent beads or bacteria. In these assays, externalized beads have been quenched with trypan blue, which reduces the fluorescence emitted by uninternalized particles [15,16]. PS beads internalization observed in the present work may be attributable to endocytosis, rather than phagocytosis. Fluorescence-based detection of internalized particles may not fully represent phagocytic activity alone and should be interpreted with caution. The BSA/Glucose cushion partially reduces the unbounded particles, while the quenching step with trypan blue does not adequately quench the fluorescence emitted by non-internalized particles. In addition, this treatment resulted in a nonspecific reduction in fluorescence emitted by both internalized and non-internalized beads. These findings suggest that trypan blue may affect fluorescence intensity independently of particle localization, potentially leading to a misinterpretation of internalized particles. In addition, trypan blue is highly toxic to both cells and operators. Taken together, these observations indicate that conventional bead-based assays may overestimate phagocytic activity and reduce measurement accuracy.

Therefore, over the last few years, phagocytosis studies in mammals have begun to exploit the recently developed pH-sensitive dyes, which emit fluorescence at specific phagolysosome pH [18,30]. pH-sensitive dyes represent a safer, more selective, and sensitive alternative, allowing the discrimination between internalized and membrane-bound particles without the need for chemical quenching. Moreover, a pH-sensitive phagocytosis assay showed signal emission from solely internalized bacteria, regardless of the in vitro conditions used for the phagocytosis assay. This observation is consistent with previously published manuscripts, validating pH-sensitive phagocytosis assays on aquatic species, namely Nile tilapia (*Oreochromis niloticus*), Atlantic salmon (*Salmo salar*), blue mussels (*Mytilus edulis*) [17], rainbow trout (*Oncorhynchus mykiss*) [8,31], and Mediterranean mussel (*Mytilus galloprovincialis*) [24]. Collectively, these studies support the applicability and robustness of pH-sensitive probes for phagocytosis assessment across phylogenetically distant aquatic organisms. The higher specificity of these probes may be particularly valuable when comparing subtle immunological differences among experimental groups.

A key aspect to consider in phagocytosis assays is the ability of phagocytic cells to recognize exogenous microorganisms, which is enhanced by opsonization. Opsonins specifically bind bacterial antigens, facilitating their efficient recognition, attachment, and internalization by the phagocytes [32,33,34]. Thus, opsonization represents a crucial step in improving phagocytic efficiency and more closely mimicking physiological immune responses. Some authors demonstrated that phagocytic behavior in rainbow trout IgM+ B cells was significantly improved by previous bacterial opsonization [35]. This important innate capacity appears to be evolutionarily conserved across vertebrate cell subsets [32,36]. To the best of our knowledge, the opsonization step has not yet been incorporated into phagocytosis assays in aquatic species, highlighting a potential gap in current methodologies. Therefore, this study investigates the effects of three plasma concentrations to assess the best experimental condition for rainbow trout leukocyte phagocytosis assays. Due to the lack of published literature on fish, we selected the most exploited concentrations in mammalian phagocytosis assays: 10%, 30%, and 60% [16,37,38]. This approach allowed us to evaluate the dose-dependent effect of opsonization on phagocytic activity and to identify optimal experimental conditions for future studies in teleostean immunology. The results obtained demonstrated that opsonization sharply increased the percentage of trout phagocytic leukocytes, consistent with previously published works [16,35,38,39]. Moreover, in rainbow trout leukocytes, 30% and 60% autologous plasma concentrations resulted in higher fluorescence intensity compared to non-opsonized samples and to 10% plasma, which is directly proportional to the number of phagocytosed bacteria. From a practical perspective, 30% plasma may represent a suitable compromise between assay performance and plasma availability.

The second set of experiments demonstrated that both temperature and incubation time significantly affect the performance of the pHR-based phagocytosis assay in rainbow trout PBLs. A higher percentage of phagocytic cells was observed at 16 °C, regardless of incubation time. In comparison, a 2-h incubation at 16 °C resulted in a significantly greater number of phagocytic cells and higher fluorescence intensity compared to the other tested conditions. These results suggest that phagocytic activity in rainbow trout PBLs is strongly influenced by temperature, with an optimum temperature corresponding to the fish’s rearing conditions. This finding is consistent with the physiological characteristics of ectothermic organisms, in which cellular processes, including innate immune functions, are modulated by environmental temperature [21,40,41]. Taken together, these observations suggest that, although heterotherm phagocyte activity can be analyzed across a variable temperature range, measurements made at 16 °C more accurately represent their natural milieu *in vitro.* This is consistent with the natural physiology of rainbow trout and with other studies measuring phagocytic activity in different heterotherm species, emphasizing the importance of adopting biologically relevant assay temperatures when comparing immune responses among studies [21,42,43]. Flow cytometric analyses further supported and extended the microscopy observations, confirming the overall trend in phagocytic myeloid cells under the tested conditions. In particular, the higher-throughput nature of this approach enabled rapid analysis of a substantially larger number of cells, providing a more robust assessment of phagocytic responses across experimental conditions [8,30,31]. Minor discrepancies between the two methods may be explained by flow cytometry’s higher sensitivity and its ability to capture rare events [17]. Indeed, in this study, the phagocytic lymphoid cells, detected by flow cytometry, represented 0–4% of the total subpopulation and exhibited 10 times lower fluorescence than myeloid cells. In contrast, by conventional fluorescence microscopy, the signal was exclusively detected in myeloid cells. Further experiments are needed to determine whether this methodological difference reflects different instrument sensitivity or a null or lower degree of acidification in lymphoid cells. However, the combined use of microscopy and flow cytometry provides complementary insights, with microscopy offering visual resolution and flow cytometry enabling high-throughput and quantitative characterization of cell-specific responses [8]. Therefore, these findings highlight the importance of methodological standardization in phagocytosis assays.

From an applied perspective, the implementation of this optimized and standardized phagocytosis assay may provide a reliable tool for routine immune monitoring in aquaculture, supporting early disease detection and the evaluation of management strategies aimed at improving fish health and welfare.

## 5. Conclusions

This study highlights the importance of standardization in phagocytosis assays for aquatic species, demonstrating that experimental variables, including opsonization, temperature, and incubation time, can significantly influence outcomes. The use of pH-sensitive probes represents a reliable and safer alternative to conventional approaches, enabling more selective detection of internalized particles without the need for washing or quenching steps. Experimental conditions identified in this work, including plasma-mediated opsonization and incubation time and temperature, provide a robust framework for *in vitro* phagocytosis assays in rainbow trout. These optimized conditions may facilitate more reproducible comparisons among studies and improve the interpretation of immune functional data, contributing to early disease detection and improved management practices for fish health and welfare. Overall, these findings improve the consistency and interpretability of phagocytosis measurements in teleost immunology and pave the way for standardized procedures at the highest level across different laboratories and countries.

## Data Availability

The original contributions presented in the study are included in the article; further inquiries can be directed to the corresponding author.

## Supplementary Materials

The following supporting information can be downloaded at: https://www.mdpi.com/article/10.3390/ani16121760/s1, Figure S1: Representative microscopic images showing bacteria-based phagocytosis assays used to study fish innate immune functions. (a) Brightfield microscopic image showing trout leukocytes and bacteria. (b) Fluorescence microscopic image highlighting phagocytosed pHrodo-*E. coli* (green) and the absence of fluorescence emission in the non-phagocytosed *E. coli.*

## Abbreviations

The following abbreviations are used in this manuscript:

PBLs	Peripheral Blood Leukocytes
pHR	pHrodo™ Green *E. coli* BioParticles™ Conjugate for Phagocytosis
Cyp-Her 5E	4-{2-[(1E,3E,5E)-5-(3-{6-[(2,5-dioxopyrrolidin-1-yl)oxy]-6-oxohexyl}-3-methyl-5-sulfo-1,3-dihydro-2Hindol-2-ylidene)penta-1,3-dienyl]-3,3-dimethyl-5-sulfo-3H-indolium-1-yl}butane-1-sulfonate
TPE-Cy	Tetraphenylethylene-cyanine
21G	21 Gauge needle
Li-Heparin	Lithium-Heparin
DPBS	Dulbecco’s modified Phosphate-Buffered Saline
L15	Leibovitz’s 15 medium
FBS	Fetal Bovine Serum
BSA	Bovine Serum Albumin
DAPI	4′,6-diamidino-2-phenylindole
RFU	Relative Fluorescence Unit
IQR	Inter-Quartile Range
