# Supplementary material for: Setup of a Simple and Cost-Effective pH-Sensitive Assay to Evaluate Phagocytosis in Rainbow Trout (Oncorhynchus mykiss) Peripheral Blood Leukocytes
Source: Animals (Basel). 2026 Jun 6;16(12):1760. doi: 10.3390/ani16121760 (PMC13295431; doi:10.3390/ani16121760)
Supplement: Supplementary file 1 [file animals-16-01760-s001.zip › animals-4326824-supplementary.pdf]

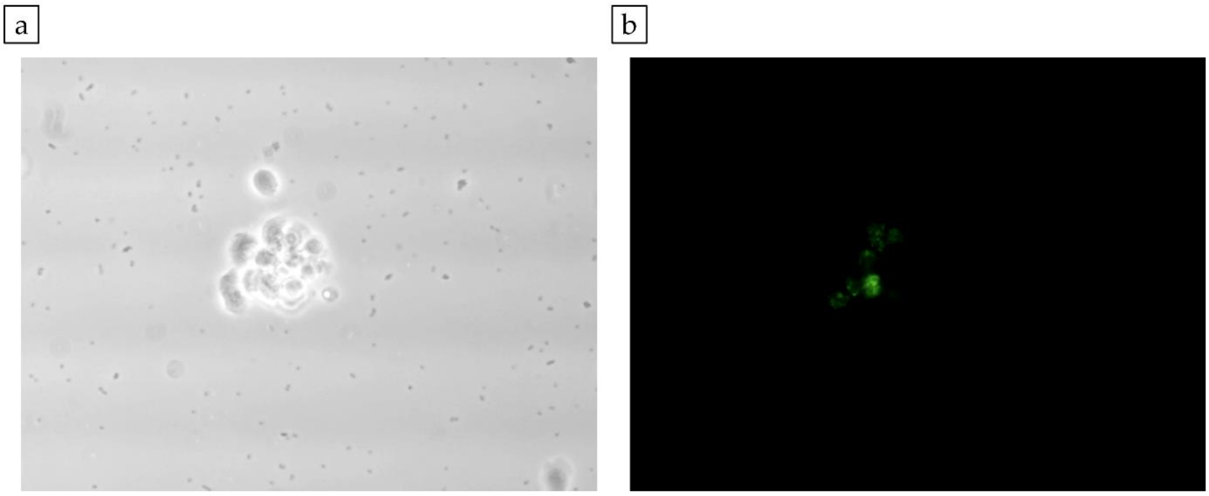

Figure S1. Representative microscopic images showing bacteria-based phagocytosis assays used to study fish innate immune functions. (a) Brightfield microscopic image showing trout leukocytes and bacteria. (b) Fluorescence microscopic image highlighting phagocytosed pHrodo-*E. coli* (green) and the absence of fluorescence emission in the non-phagocytosed *E. coli.*
